# Recombinant Enterovirus 71 Viral Protein 1 Fused to a Truncated Newcastle Disease Virus NP (NPt) Carrier Protein

**DOI:** 10.3390/vaccines8040742

**Published:** 2020-12-07

**Authors:** Suhaili Mustafa, Noraini Abd-Aziz, Wuan-Ting Saw, Sien-Yei Liew, Khatijah Yusoff, Norazizah Shafee

**Affiliations:** 1 Department of Animal Science and Fishery, Universiti Putra Malaysia, Bintulu Sarawak Campus, Bintulu 97008, Malaysia; 2 Centre for Virus and Vaccine Research (CVVR), School of Science and Technology, Sunway University, Subang Jaya 47500, Malaysia; norainiaa@sunway.edu.my; 3 Department of Microbiology, Universiti Putra Malaysia, Serdang 43400, Malaysia; swting0502@hotmail.com (W.-T.S.); sienyei86@gmail.com (S.-Y.L.); kyusoff@upm.edu.my (K.Y.); nshafee@gmail.com (N.S.)

**Keywords:** enterovirus 71, recombinant vaccine construct, hand foot and mouth disease, nucleocapsid protein

## Abstract

Enterovirus 71 (EV71) is the major causative agent in hand, foot, and mouth disease (HFMD), and it mainly infects children worldwide. Despite the risk, there is no effective vaccine available for this disease. Hence, a recombinant protein construct of truncated nucleocapsid protein viral protein 1 (NPt-VP1_198–297_), which is capable of inducing neutralizing antibody against EV71, was evaluated in a mouse model. Truncated nucleocapsid protein Newcastle disease virus that was used as immunological carrier fused to VP1 of EV71 as antigen. The recombinant plasmid carrying corresponding genes was constructed by recombinant DNA technology and the corresponding protein was produced in *Escherichia coli* expression system. The recombinant NPt-VP1_198–297_ protein had elicited neutralizing antibodies against EV71 with the titer of 1:16, and this result is higher than the titer that is elicited by VP1 protein alone (1:8). It was shown that NPt containing immunogenic epitope(s) of VP1 was capable of inducing a greater functional immune response when compared to full-length VP1 protein alone. It was capable to carry larger polypeptide compared to full-length NP protein. The current study also proved that NPt-VP1_198–297_ protein can be abundantly produced in recombinant protein form by *E. coli* expression system. The findings from this study support the importance of neutralizing antibodies in EV71 infection and highlight the potential of the recombinant NPt-VP1_198–297_ protein as EV71 vaccine.

## 1. Introduction

Enterovirus 71 (EV71), a human virus that belongs to family *Picornaviridae* [1], has been extensively studied, because it is a causative agent of hand, foot, and mouth disease (HFMD) and it is associated with severe neurological complications [2]. It was first isolated as the etiological agent of HFMD from a young child in California, United States in 1969 [3]. Enteroviruses, such as Enterovirus 71 (EV−A71), Coxsackievirus type A16 (CV-A16), and other HFMD-causing entroviruses have caused more than seven-million infections, including 2457 fatalities in China from 2008 to 2012. Most HFMD infections that led to fatality were due to the EV-A71 virus [1]. Since 1997, EV71 has mainly circulated in Asia Pacific region and most of the countries in this region are developing countries, such as Malaysia, China, Taiwan, Brunei, and Vietnam. The most promising vaccine candidate is currently being tested while using inactivated whole EV71 virus [4]. Other candidates of inactivated whole virus vaccines are also being studied [5,6]. Besides using the whole virus, virus-like particles [7] or viral protein subunit vaccines [8] are also being tested. EV71 viral proteins that were expressed in either bacterial or plant system [9,10,11] and synthetic peptides harboring antigenic epitopes of EV71 viral proteins [12,13] serve as alternatives for EV71 vaccine production. However, there are several drawbacks of using inactivated whole-virus or live-attenuated virus as a vaccine, such as its biosafety issues, cost of production, tedious procedure of viral preparation, as well as the difficulty of storage and delivery. This has led to the development of other types of vaccines, including epitope peptide vaccine, consisting of the specific immunogenic epitope, which is capable of stimulating an immune response.

The recombinant protein technology enables polypeptide-based vaccine to be produced in bulk, irreversible of their virulent form, easier, and cheaper. Administering the immunogenic protein from an infectious pathogen produces a more targeted and specific immune response. In addition, the risk of active infection that is caused by live attenuated vaccines or inactivated virus vaccines can also be reduced. Despite the specificity, the small polypeptide is often relatively less immunogenic and requires the use of delivery systems, such as carriers or adjuvants, in order to boost up the immunogenicity of the epitope peptide. Nucleocapsid protein (NP) of Newcastle disease virus (NDV) is the most abundant protein on the viral particle [14]. It heterodimerizes in order to form a herringbone structure, a classical morphology of Paramyxoviruses. Recombinant NP are still able to assemble into this herringbone or ring-like structure when expressed in baculovirus [15] and *E. coli* expression systems [16]. Additionally, this structure can still be formed by the NP, even though there is a small peptide added onto its C-terminal [16]. It has been shown that such a fusion protein containing a C-terminal foreign peptide was exposed on the surface of the NP particles [17,18]. When the HN and F protein fragments were fused to the C-terminal end of the NP, they were able to induce high immune responses in chickens [19]. These results showed the potential use of NP as a carrier protein for foreign immunogens.

In the previous study, a truncated nucleocapsid protein (NPt), which was derived from the NDV, was used as a carrier protein to deliver the first 100 amino acid of the N-terminal of VPI (VP1_1–100_) into animals. The obtained results showed that the recombinant protein exhibited strong immunogenic properties in rabbits and mice [20,21,22]. However, the newborn mice were only partially protected upon the lethal challenge of EV71. The partial protection was likely due to the insufficient antigenic properties of the N-terminal end of VP1 antigen. Neutralizing epitopes on the VP1 protein of EV71 were shown to be located at its C-terminal hydrophilic moiety [12]. Synthetic peptides, designated as SP70 and SP55, which corresponded to amino acids 163–177 and 208–222 of this C-terminal of VP1, respectively, were capable of inducing strong immune responses against EV71 in mice [13]. Based on this information, it is hypothesized that hydrophilic fragments of the C-terminal region of EV71 will be able to be expressed as fusions to carrier molecules without affecting its antigenicity. Therefore, in the present study, the antigenicity of the recombinant plasmids carrying the NPt gene as a fusion to VP1 gene regions encoding for VP1_198–297_ peptides were evaluated in the mice model. 

## 2. Materials and Methods 

### 2.1. Synthesis and Amplification of VP1_198–297_ Gene by Polymerase Chain Reaction (PCR)

The primer pair was designed for the synthesis of each gene fragment of VP1_198–297_ based on the nucleotide sequences of the VP1 of EV71 strain MY104/9/SAR/97 (accession no.: AF). DNA sequencing analysis of positive clones was conducted by Macrogen Sequencing Service (Korea). The pTrcHis2-NPt-VP1_1–100_ and pET30a-VP1fl plasmids constructs were extracted using the HiYield plasmid mini kit (Yeastern Biotech, Taipei, Taiwan), according to the manufacturer’s instruction. The synthesis of a gene fragment of VP1 was carried out by PCR. The reaction was performed in 50 µL reaction mixtures containing 1 U DyNAzyme EXT DNA polymerase, 1 X DyNAzyme EXT buffer [50 mM Tris-HCl, 15 mM (NH_4_)_2_ SO_4_, 0.1% Triton X−100; pH 9.0], 1.5 mM MgCl_2_, 200 µM dNTPs, 0.6 µM forward and reverse primers, and 1 ng of pET30 a-VP1fl plasmid as template. The mixtures were incubated in a Mastercycler^®^ thermal cycler (Eppendorf, Enfield, CT, USA) with a heated lid. The reactions were initiated with pre-denaturation of the DNA template (94 °C, 3 min.), followed by a 35-cycle PCR profile: denaturation (94 °C, 30 s), annealing (60 °C, 1 min.), and extension (72 °C, 1 min.). The reaction was finished with a final extension step (72 °C, 7 min.).

### 2.2. Purification and Transformation of PCR Products

The PCR product was separated by electrophoresis in big wells of a 1% (*w/v*) TAE agarose gel. The purified VP1 DNA fragments and the pTrcHis2-NPt-VP1_1–100_ plasmid were separately digested in 20 µL of restriction enzyme mixture containing 5–10 U *Sna*BI, 1X Tango^TM^ buffer. The digested products were separated by agarose gel electrophoresis and purified while using the Gel Extraction Kit (Qiagen, Germantown, MD, USA), according to the manufacturer’s instruction. The VP1 DNA fragments were inserted into *Sna*BI and *Eco*RI sites of pTrcHis2-NPt plasmid separately with the ratio 1:3 in a 20 µL of ligation mixtures containing 2.5 U T4 DNA Ligase (Fermentas, Waltham, MA, USA) and 1X T4 DNA Ligase buffer and then incubated at 4 °C for 16 h. 

Competent cells of *E. coli* TOP10 were prepared while using the calcium chloride (CaCl_2_) method. The ligation product was transformed into the competent *E. coli* TOP10 cells using the heat-shock method [23]. This was followed by the addition of 1 mL of Super Optimal broth with catabolite repression (SOC medium) to the mixture and incubated at 37 °C for 1 h with an agitation rate at 250 rpm. Approximately 50–200 µL of each transformation mixture was spread onto LB agar plates containing 50 µg/mL of ampicillin and incubated overnight at 37 °C. Ten pure single colonies of positive transformants were picked for the screening of successful transformations. Each colony was individually inoculated in 10 mL of LB broth containing 50 µg/mL of ampicillin. After overnight incubation, 3 mL of the cultures were used for plasmid extraction using the HiYield plasmid mini kit (Yeastern Biotech, Taipei, Taiwan) according to the manufacturer’s instruction. PCR, restriction enzyme reaction, and DNA sequence analysis confirmed the orientation of inserts.

### 2.3. Expression, SDS-PAGE and Western Blot of Recombinant NPt-VP1_198–297_ Proteins

The positive clone was inoculated in 5 mL LB medium containing 50 µg/mL of ampicillin overnight at 37 °C in a shaking incubator. One milliliter of overnight cultures was inoculated in 100 mL of fresh LB medium containing 50 µg/mL of ampicillin. When the OD600 nm of cultures reached 0.6 to 0.8, isopropyl-β-D-thiogalactopyranoside (IPTG) was added to a final concentration of 1 mM. Immediately following the addition of IPTG, 1 mL of cultures were collected and left as 0 h induction sample. After that, the remaining cultures were further incubated, and 1 mL of culture was collected at every hour until 8-h incubation. All of the samples were centrifuged at 6000× *g* in Sorvall Legend Micro 17 microcentrifuge (Thermo Scientific, Waltham, MA, USA) at 4 °C. The collected cell pellets were then resuspended in 100 µL of sterile phosphate buffer saline (PBS) and analyzed by 12% SDS-polyacrylamide gel electrophoresis (SDS-PAGE) and western blotting with anti-His, anti-NDV, and anti-VP1 antibody. The intensities of protein bands were quantified by ImageJ software (National Institutes of Health, Bethesda, MD, USA). The expression level was expressed by the ratio of the readings of band intensities at each time-point divided by the reading of band intensities at 1-h post-induction. For the scale-up expression of recombinant proteins, the volumes of each culture were increased from 0.5 L to 2 L and the samples were harvested after 5 h post-induction with 1.0 M IPTG (Table 1).

### 2.4. Purification of Recombinant Protein

Bacterial culture (2 L) expressing recombinant protein was harvested by centrifugation at 6500× *g* at 4 °C for 10 min. and washed with ice-cold 1X PBS and resuspended in 0.03 volume of lysis buffer, and then incubated at room temperature for 30 min. with gentle mixing. The cell lysate was centrifuged at 12,000× *g* at 4 °C for 20 min. The pellet was then resuspended in 0.03 volume of lysis buffer containing 1% Triton X−100 and then incubated on ice for 10 min. Subsequently, the suspension was centrifuged at 12,000× *g* at 4 °C for 20 min. This step was repeated with lysis buffer without Triton X−100. The washed pellet was resuspended in phosphate buffer [20 mM sodium phosphate, 500 mM NaCl; pH 7.4] containing 8 M urea, 0.3 mM reduced glutathione (GSH), and 5% glycerol and incubated for 2 h at room temperature with gentle shaking. After centrifugation at 12,000× *g* for 20 min. at 4 °C, the supernatant was subjected to a HisTrap HP column purification. The column was washed with five volumes of binding buffer containing 8 M urea, 5% glycerol, 3 mM GSH, 0.3 mM glutathione disulfide (GSSG), and 30 mM imidazole. The column was further washed with binding buffer. as mentioned before with a gradual decrease of urea concentration until 2 M and washed with 5 column volumes of binding buffer containing 2 M urea, 5% glycerol, 3 mM GSH, 0.3 mM GSSG, and 30 mM imidazole. After that, the target protein was eluted from the column by elution buffer. The elution product was dialyzed against sodium phosphate buffer containing 1 M of urea overnight at 4 °C and then concentrated by Vivaspin Concentrator.

### 2.5. Immunization of NPt-VP1_198–297_ in a Mouse Model

The Animal Care and Use Committee, Faculty of Veterinary Medicine, Universiti Putra Malaysia, Selangor approved all of the animal works in this study (AUP no: 10R84). The animals were raised and cared for according to The Code to Care and Use of Animals in Research. The methods of animal experiments were performed under the guidelines that were prescribed by the Malaysian Association for Accreditation of Laboratory Animal Care. Groups (n = 8) of 6–8-week-old adult female BALB/C mice were immunized with the recombinant NPt-VP1_198–297,_ NPfl (as control), VP1fl (as control) individually. Each group of mice was immunized intraperitoneally with 10 µg of purified protein (in PBS, pH 7.4), which was emulsified with 50% Freund’s complete adjuvant (Sigma, St. Louis, MO, USA). Two boosters (10 µg proteins emulsified with 50% Freund’s incomplete adjuvant) were administered to each mouse every two weeks. The blood samples were collected by bleeding of tail’s vein at weeks 0, 2, 4, 6, 8, 9, and 10. The collected blood samples were stored overnight at 4 °C to allow for the clotting of red blood cells (RBC). On the following day, sera at the upper layer were carefully pipetted out to a new, sterile tube. The remaining blood samples were centrifuged at 2000 rpm for 20 min. and the remaining sera were pipetted out carefully. 

### 2.6. Indirect Enzyme-Linked Immunosorbent Assay (ELISA)

Titers of anti-VP1 and anti-NP IgG in mice sera were evaluated by an indirect enzyme-linked immunosorbent assay (ELISA). First, each well of a flat-bottomed, high binding 96 wells ELISA microtiter plate (Greiner Bio-One, Frickenhausen, Germany) was pre-coated with 100 µL of 1.5 µg/mL VP1 or NP in 1X TBS buffer [0.1 M Tris, 0.15 M NaCl; pH 8.0], respectively. Subsequently, the plates were sealed and incubated overnight with shaking at 4 °C in the dark. The wells were coated with pET30, a control protein that served as a negative control. On the following day, the coating solution was removed by gently knocking the plates on a paper towel and washed three times with 1X TBS buffer containing 0.05% (*v/v*) Tween−20 (TBS-T) at room temperature for 5 min. The remaining buffer in wells was removed by patting the plates. After that, each well was blocked with 250 µL of blocking solution [5% (*w/v*) bovine serum albumin in TBS] for 2 h at 4 °C. Subsequently, the plates were washed three times with TBS-T. Subsequently, 100 µL of diluted serum samples (1:50) was added to the corresponding wells and the plates were incubated at room temperature for 1 h with gentle shaking. After washing the plates with TBS-T, 100 µL of anti-mouse IgG conjugated with horseradish peroxidase (Cell Signaling, Danvers, MA, USA) (1:1000 in TBS) was added into each well and then incubated at room temperature for 1 h with gentle shaking. Afterwards, the plates were washed with TBS-T for five times and then developed with 100 µL of 0.4 mg/mL o-phenylenediamine dihydrochloride substrate (Acros Organics, Carlsbad, CA, USA) in 0.05 M Citrate Phosphate buffer [Na_2_ HPO_4_, citric acid, pH 5.0] containing 20 µL fresh 30% H_2_ O_2_. After incubation at room temperature for five min., the reaction was stopped by adding 100 µL of 3 M sulphuric acid (H_2_ SO_4_). The absorbance values were then measured at 490 nm on iMark Microplate Absorbance Reader (BioRad, Hercules, CA, USA). 

### 2.7. Immunoblotting Analyses

Recombinant VP1 and NP were separated on 12% SDS-PAGE gel and then transferred onto PVDF membranes as antigen for immunoassay. After blocking with 10% milk diluent, the mice sera containing antibodies to the recombinant VP1 and NP were used as primary antibodies (1:500 in 1X TBS) in order to probe the blotted recombinant proteins. Rabbit anti-VP1 serum (1:2000 in 1X TBS) and rabbit anti-NDV serum (1:9000 in 1X TBS) were used to serve as positive controls. Subsequently, the membranes were incubated with appropriate alkaline phosphatase-conjugated secondary antibodies (Santa Cruz Biotechnology, Santa-Cruz, CA, USA) with a dilution of 1:5000 in 1X TBS at room temperature for 1 h. The mixtures of BCIP/NBT in 10 mL alkaline phosphatase buffer were added for signal development. When the desire bands appeared, the substrate was discarded, and the membrane was washed with distilled water. 

### 2.8. Neutralization Assay

A neutralization assay was carried out in order to determine the presence of neutralizing antibodies in the immunized mice sera. In brief, 1.5 × 10^4^ of Vero cells were seeded and cultured in a 96-well tissue culture plate (Corning, New York, NY, USA) with DMEM (PAA, Pittsburgh, PA, USA) containing 5% FBS (PAA, USA) at 37 °C with 5% CO_2_ for 36 h. The pooled mice sera collected from pre-immunized or immunized mice were inactivated at 56 °C for 30 min. Afterwards, each sample was two-fold serially diluted with DMEM media containing 2% FBS (PAA, USA), 1% L-glutamine and 1% antibiotic-antimycotic solution (PAA, USA). The diluted sera were mixed with an equal volume of EV71 strain A104 containing a final 1000 TCID50. After incubation at 37 °C for 2 h, the mixtures of serum and virus were added to the Vero cell culture in the designated wells (well 1:8 to 1:512). The positive control was added with EV71 post-infection serum and a well containing virus only were served as a negative control. After seven days of incubation at 37 °C with 5% CO_2_, the cytopathic effect (CPE) of cells was observed under an inverted light microscope (Olympus, Tokyo, Japan) and the neutralization titers were determined by calculating the highest dilutions that could result in less than 50% CPE. All of the experimental data in this study were analyzed while using the Student’s *t*-test and presented as mean ± standard error (SE). Differences with *p* < 0.05 were considered to be significant.

## 3. Results

### 3.1. Hydrophobicity Profiles of VP1fl and VP1_198–297_

**Figure 1:**
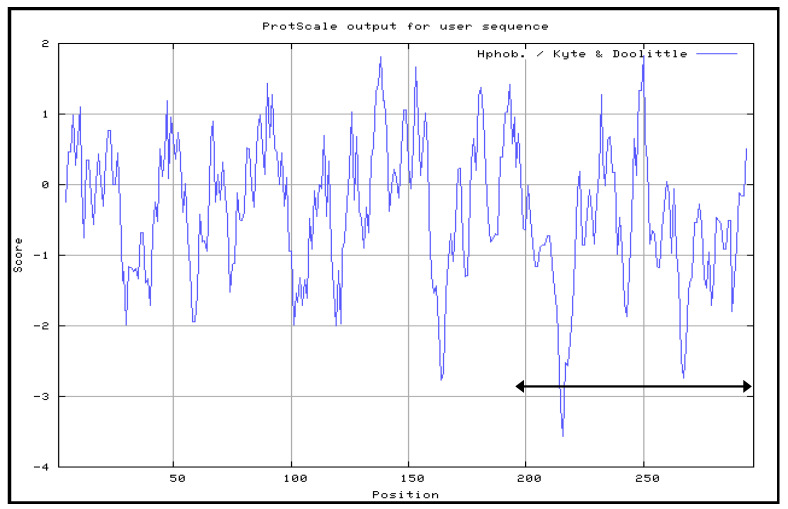
Kyte and Doolittle hydrophobicity profile plot of the VP1 capsid protein of EV71. The more positive the score is, the more hydrophobic the amino acid in that region. The amino acid position of VP1_198–297_ was indicated by double-head arrows.

**Figure 2:**
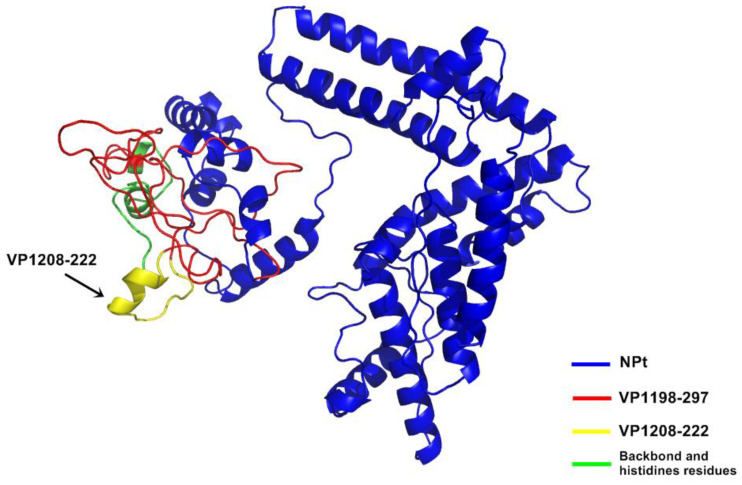
Predicted three-dimensional (3D) structure of recombinant NPt-VP1198–297 protein.

**Figure 3:**
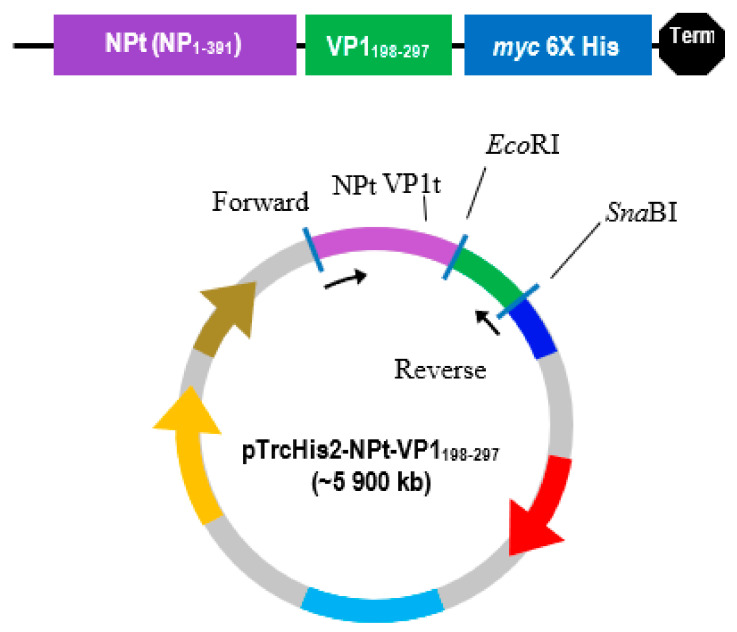
Schematic representation of the pTrcHis2 plasmid carrying a truncated nucleocapsid protein (NPt) gene of Newcastle disease virus (NDV) fused with the VP1_198–297_ gene of EV71. The gene fragment of VP1_198–297_ was synthesized by Polymerase Chain Reaction (PCR) and inserted at the C-terminal of NPt gene.

In this study, the Kyte and Doolittle hydrophobicity profile plot [24] showed that the most hydrophilic regions were located on the C-terminal of VP1 amino acid sequence (Figure 1). VP1_198–297_ contains the amino acids that were located at the major hydrophilic regions of VP1 and, thus, these were thought to be predominantly exposed on the surface of the VP1 in native. The subscript numbers following the VP1 represent the amino acid position in the full-length VP1. Scratch Protein Predictor was used for the determination of their solubility upon over expression (SOLpro), examination of continuous B-cell epitopes (CODEpro) and disulfide bridges (DIpro) as well as antigenicity (ANTIGENpro). The predicted SD structure of recombinant NPt-VP1_198–297_ protein (Figure 2 and Figure 3) predicted the immunogenic VP1 protein fragment–VP1_198–297_ was exposed on the surface of the construct, and the region bearing neutralizing epitope–VP1_208–222_ was located at the outermost of the surface.

### 3.2. Amplification of the Genes that Encode for VP1_198–297_

**Figure 4:**
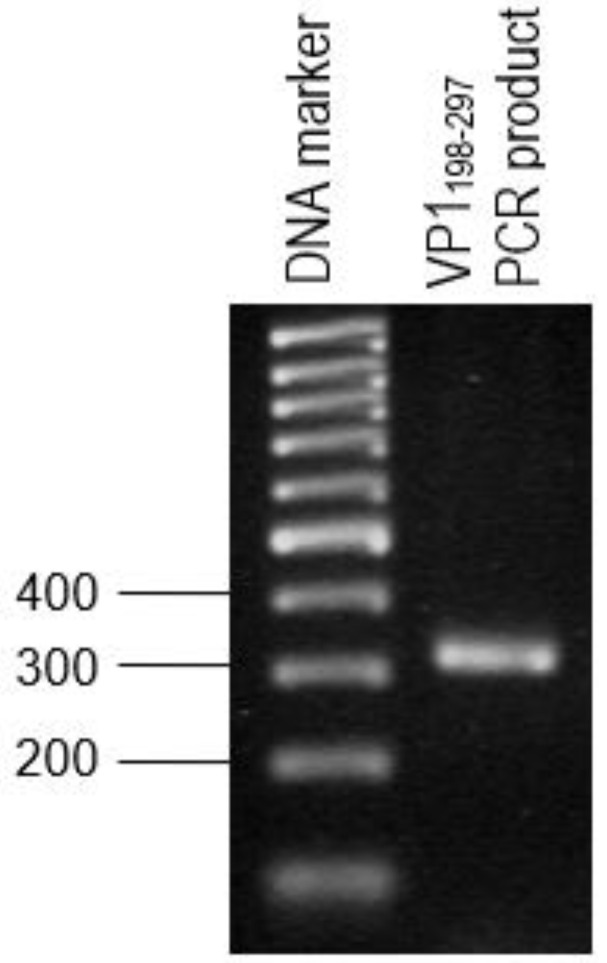
PCR product of VP1_198–297_ gene with the size of 300 bp approximately.

**Figure 5:**
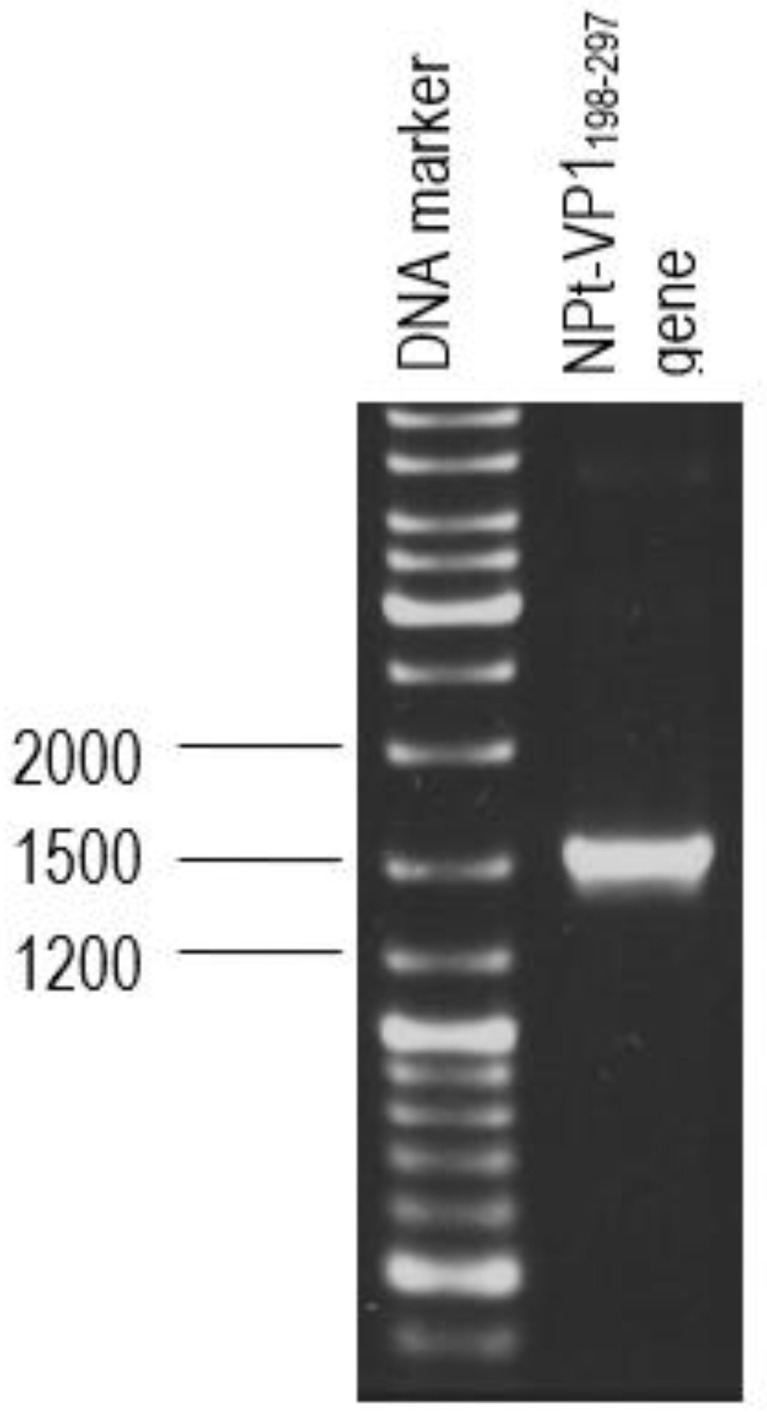
The determination of the inserts of positive clones by PCR. PCR products of the NPt harboring truncated VP1 genes using the forward primer of pTrcHis2 and the reverse primers of respective inserts.

**Figure 6:**
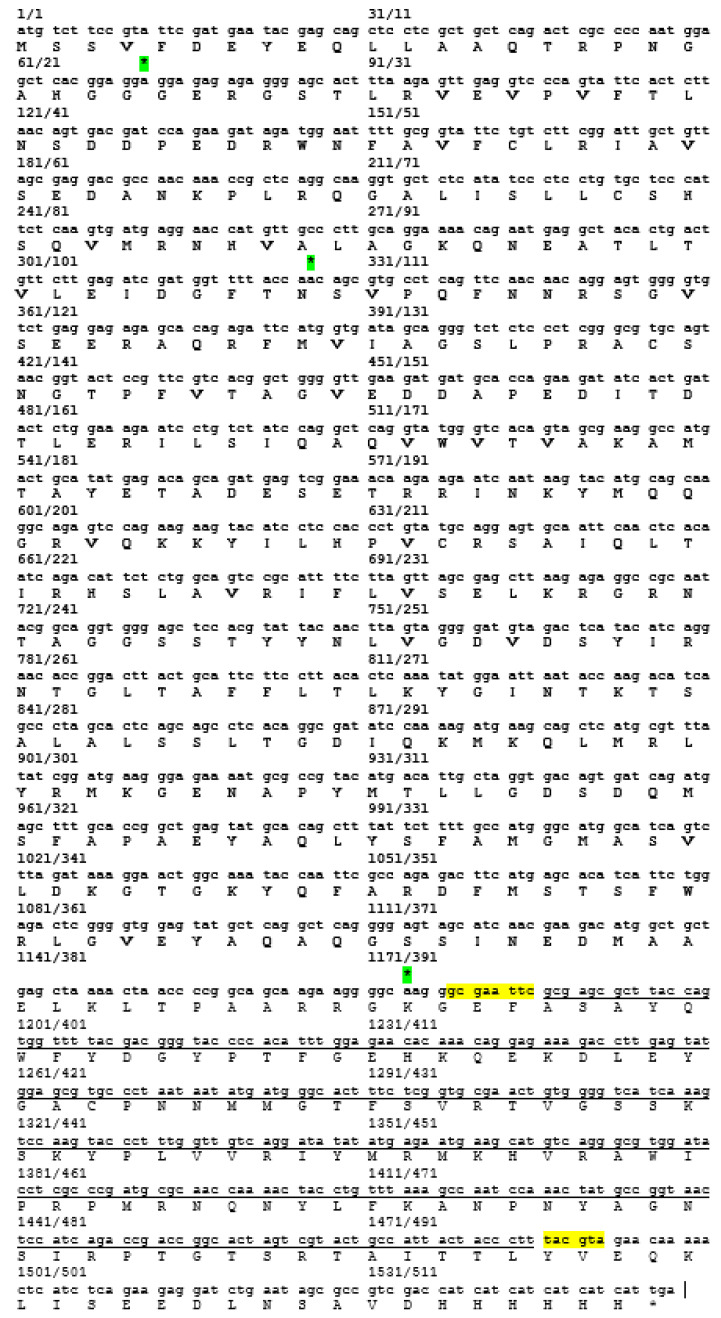
Nucleotide and amino acid sequence of gene encoding for NPt-VP1_198–297._ Black color sequence from 1–1177 nucleotide is a NP sequence and VP1_198–297_ nucleotide sequence indicates with underline ___ from 1185–1485 seq. The * indicating mutated nucleotide and highlighted text is the restriction enzymes.

**Figure 7:**
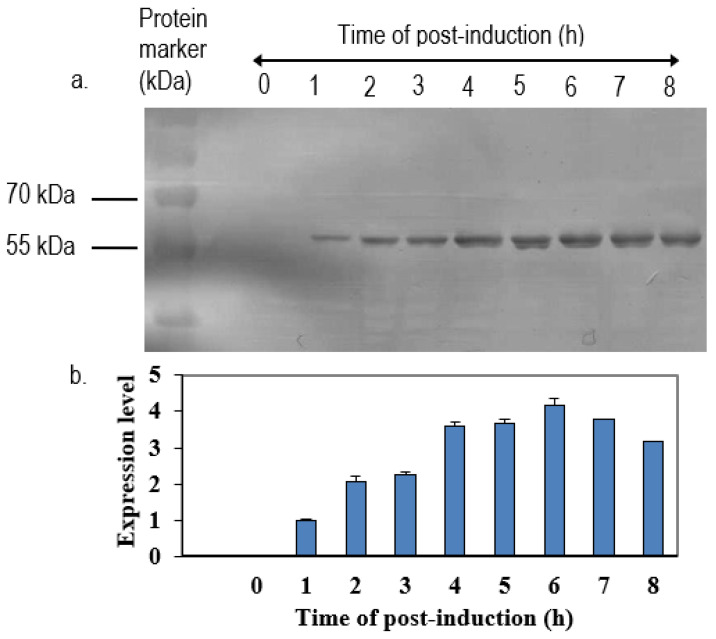
Time-course expression of the NPt-VP1_198–297_ in *E. coli* TOP10 after induction of IPTG. The cultures were collected at time points of 0 h, 1 h, 2 h, 3 h, 4 h, 5 h, 6 h, 7 h, and 8 h post-induction with IPTG. (**a**) Immunoblotting of expressed protein by anti-His monoclonal antibody. (**b**) The expression level of recombinant proteins was detected from the immunoblotted bands of expressed proteins by using ImageJ software. The expression of protein reached the peak level on 6 h post-induction.

VP1_198–297_ gene fragments (Figure 4) were successfully synthesized by PCR while using specific primers were verified on the TAE agarose gel at the sizes slightly larger than expected sizes of 300 bp, due to the presence of restriction enzyme cutting sites and extra nucleotides for efficient enzymes function. The DNA fragment was designed in order to contain *Eco*RI restriction site at the 5′ end of DNA fragments and *Sna*BI restriction site at the 3′ termini. Subsequently, the PCR product was successfully inserted into plasmid pTrcHis2 carrying gene encoding for NPt. PCR is known to be a highly sensitive method for gene amplification [25]. Even minute amounts of DNA can still be amplified while using this method. The amplification reaction was shown to produce the expected PCR products of NPt-VP1_198–297_ at the sizes of 1600 bp (Figure 5). This result indicated the truncated VP1 gene fragments were successfully inserted into pTrcHis2 plasmid carrying NPt gene (pTrcHis2-NPt) and in a correct orientation. DNA sequencing analysis further confirmed the insertion of VP1_198–297_ gene fragments into pTrcHis2-NPt plasmids at the sites between *Eco*RI and *Sna*BI in the correct orientation [12]. Figure 6 shows the nucleotide sequence, as well as amino acid sequence. The Recombinant NPt-VP1_198–297_ proteins were produced with the induction of 1 mM of IPTG under the control of *trc* promoter [26]. Upon the addition of IPTG, the expression of these recombinant proteins (Figure 7) was induced as early as 1 h post-induction. These expressed proteins contain six histidine [27] amino acid residues at the C-terminal and can be detected by anti-His antibody. The recombinant proteins were observed at the size of approximately 56.3 kDa.

### 3.3. Detection of Recombinant NPt-VP1_198–297_ Protein 

**Figure 8:**
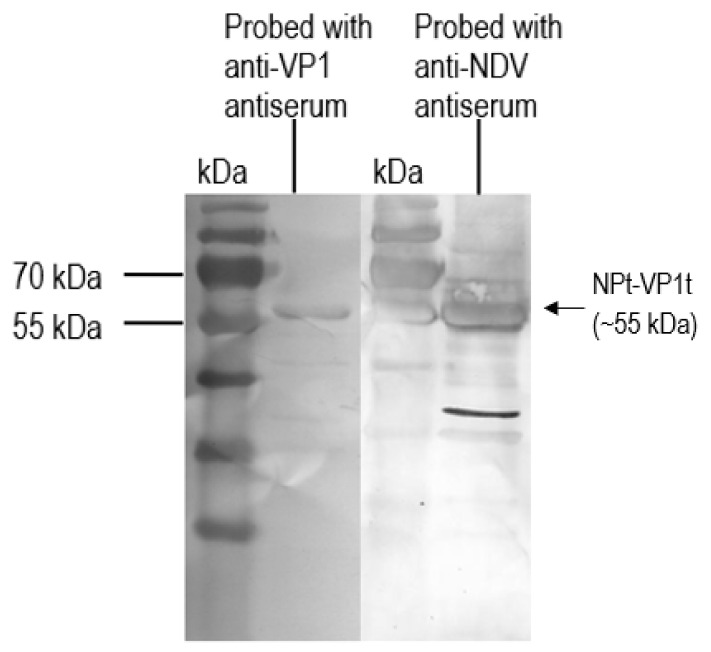
Detection of recombinant NPt-VP1_198–297_ protein with anti-VP1 (lane 1) and anti-NDV (lane 2) rabbit antiserum.

**Figure 9:**
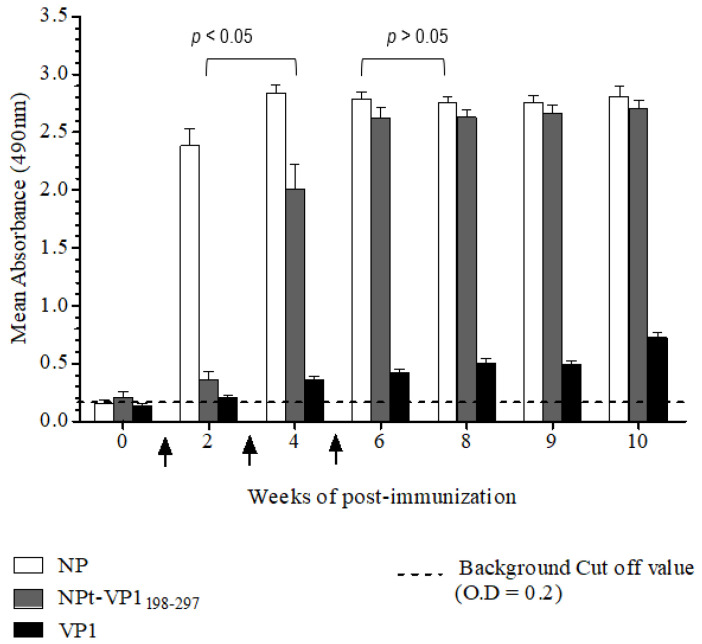
Determination of total IgG titers of anti-NP in the immunized sera by ELISA. Immunization time-points were indicated by arrows and the cut-off value was indicated by broken lines. The anti-VP1 IgG titers for NPt-VP1_197–297_ (n = 8) significantly increased (*p* < 0.05) after primary immunization and was further enhanced by every booster injection when compared to the VP1.

The purified recombinant proteins were analyzed by western blot before these proteins were subjected for the immunogenicity studies. The recombinant NPt-VP1_198–297_ protein was able to be detected by anti-NDV antibodies and anti-VP1 antibodies (Figure 8). It indicated the presence of immunogenic epitopes within the region of VP1, where the amino acid position from 198 to 297. These purified proteins were used for the immunogenicity studies while using the mouse model. The total IgG level against full-length viral protein 1 (VP1fl) and full-length nucleocapsid proteins (NPfl) were analyzed by ELISA in order to determine the antibody responses that were elicited by NPfl, NPt-VP1_198–297_, and VP1fl. The detected readings on the wells of pre-immune sera were served as the background cut-off values. All of the recombinant proteins, except VP1fl, induced high antibody responses against NPfl and maintained at a high level until week−10 post-immunization (Figure 9) with (*p* < 0.05). It indicated that the recombinant NPt-VP1_198–297_ protein is highly immunogenic.

### 3.4. Determination of Total IgG Titers of Anti-NP in the Immunized Sera

**Figure 10:**
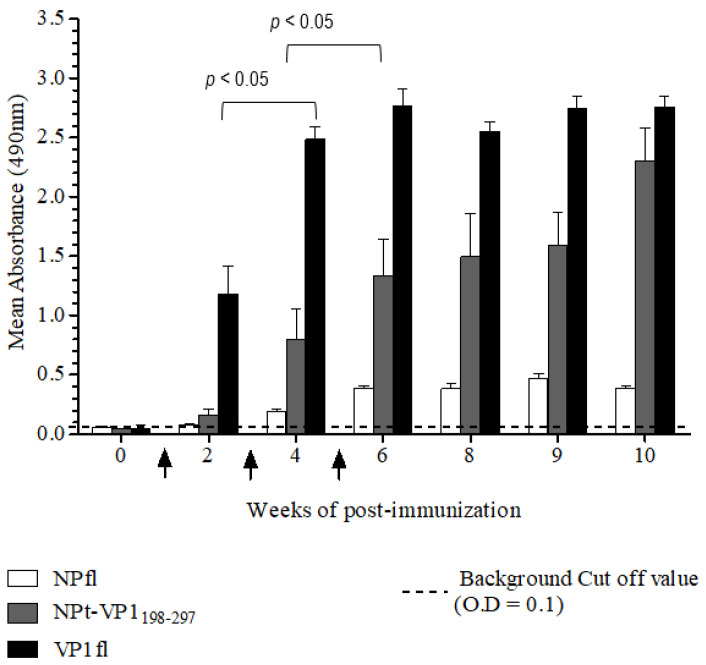
The determination of total IgG titers of anti-VP1 in the immunized sera by ELISA. Immunization time-points were indicated by arrows and the cut-off value was indicated by broken lines. The recombinant NPt-VP1_198–297_ (n = 8) proteins elicited a significant IgG level (*p* < 0.05).

**Figure 11:**
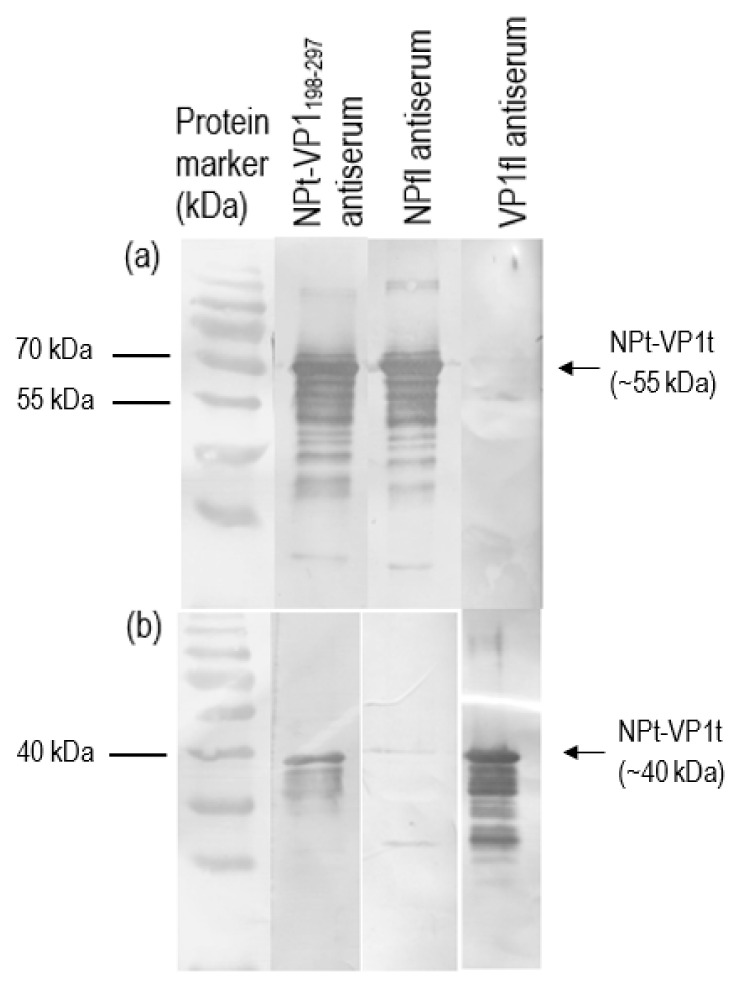
Western blot analysis using antisera of NPt-VP1_198–297_ protein against (**a**) NPfl and (**b**) VP1fl antigens. Each antiserum was diluted with 1X TBS in 500 X dilution and incubated with the antigen-coated membranes for 1 h.

The first injection of VP1fl proteins (positive control) had elicited the immune response against VP1fl proteins and the response reached the peaks after the second booster (Figure 10). The IgG level was maintained until week−10 post-immunization. The recombinant NPt-VP1_198–297_ proteins elicited a significant IgG level (Figure 10). The primary immunization of NPt-VP1_198–297_ proteins did not induce a significant response against VP1 antigen, but its response had elicited after the first booster after two weeks. The IgG level that was induced by NPt-VP1_198–297_ proteins was continually increased after two boosters until week−10 post-immunization (*p* < 0.05). Its IgG level was comparably high to that of VP1fl at week−10 post-immunization. The immunoreactivity of the recombinant NPt-VP1_198–297_ against NPfl and VP1fl was also assayed by Western blotting analysis. The pattern of immunoreactivity of the antisera that was shown in Western blot analysis was the same as that of ELISA results. All antisera, except for that of VP1fl, reacted with the capturing proteins, NPfl, and revealed a band at a molecular weight of 55 kDa (Figure 11). In contrast, the antisera obtained from the mice immunized with NPt-VP1_198–297_ and VP1fl showed reactivity against VP1fl (Figure 11). A band of 40 kDa in molecular weight was produced when the antisera were incubated with VP1fl that were coated on the membrane. The results were consistent with the results of ELISA. It was found that no antibodies reacted with NPfl or VP1fl in the pre-immune sera. There was also no immunoreactivity between the NPfl antiserum and VP1fl, and between the VP1fl antiserum and NPfl.

### 3.5. Neutralization Analysis of Immunized Mice Sera

**Figure 12:**
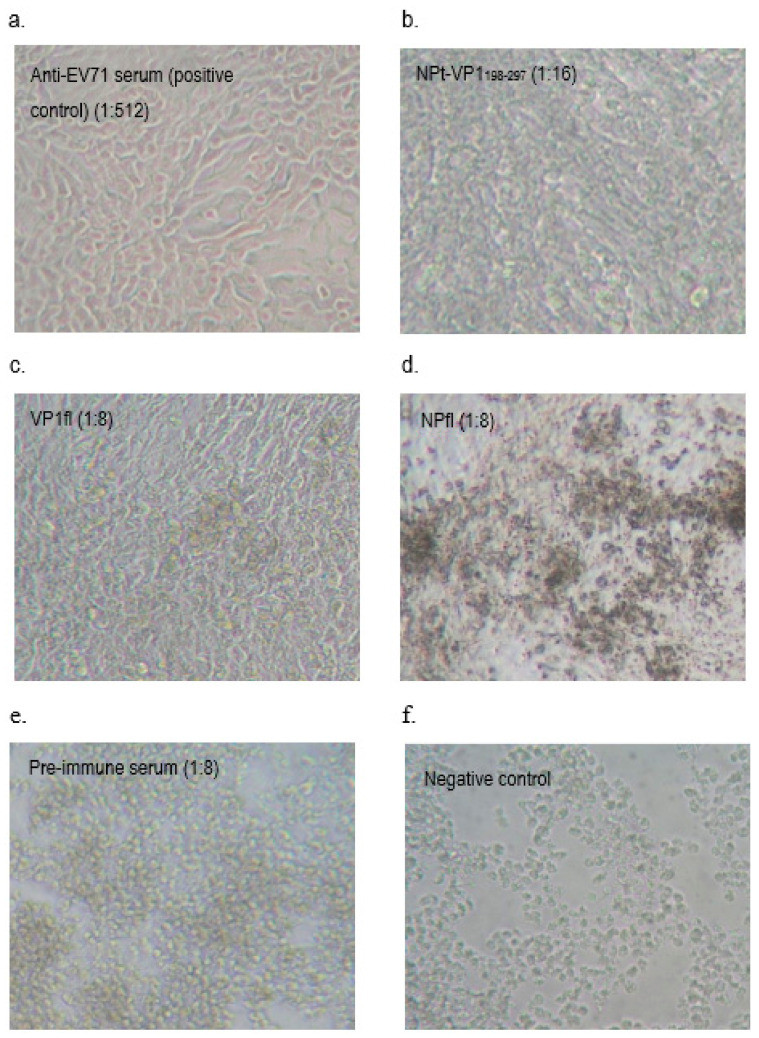
Neutralization analysis of immunized mice sera. Cytopathic effects in Vero cells following a neutralization test. Mixtures of sera dilutions and EV71 strain A104 virus were assayed on Vero cells. The cytopathic effects were examined after seven days of incubation and neutralization titers were determined. (**a**). 1:512 dilution was used for the post-challenge and the positive control sera. (**b**). NPt-VP1_198–297_-immunized samples (1:16 dilution). (**c**). VP1fl-immunized sample with 1:8 titer. (**d**,**e**). Pre-immune sera and the NPfl-immunized samples barely detectable (1:8 dilution). (**f**). No serum was used in the negative control samples. Magnification = 200X.

The antisera from immunized mice were analyzed by in vitro microneutralization assay in order to determine whether the elicited antibodies were capable of neutralizing live EV71 strain A104 from the infection to Vero cells. All of the collected sera from pre-immunized and immunized mice were pooled together based on the group. The pooled mice sera collected from pre-immunized or immunized mice were inactivated at 56 °C for 30 min. and mixed with EV71 strain A104. The antiserum, which was collected from the mouse-adapted EV71 P5-infected mice, served as a positive control, and it exhibited a neutralization titer of 1:512. The antisera obtained from the mice immunized with NPt-VP1_198–297_ and VP1fl showed the neutralizing activities against EV71. A better neutralization effect at titer of 1:16 was exhibited by the antiserum raised against NPt-VP1_198–297_ proteins when comparing the VP1fl (1:8) and other antiserum, which resulted in a neutralization titer less than 1:8. The EV71-neutralizing antibodies in the pre-immune serum and antisera raised against NPfl were barely detectable (<1:8). It can be observed that the anti-NPt-VP1_198–297_ antiserum prevented Vero cells from CPE (Figure 12a) as compared to NPfl, where we can see the massive changes on the cell morphology that cannot prevent the CPE (Figure 12d). This observation shows that recombinant NPt-VP1_198–297_ was able to induce a significant neutralizing immune response against EV71 when compared to the others. (Figure 12 and Table 2). The experiment was conducted in three replicates and for sera immunized with NPt-VP1_198–297_, showing a significant different (*p* < 0.05) when compared to VP1fl and NPfl. 

## 4. Discussion

The Kyte and Doolittle hydrophobicity profile was used in order to examine the VP1fl in the present study. Hydrophilic regions of a protein are known to be exposed on the surface of the protein when it is in its tertiary conformation [28]. It would be correlated between antigenic sites and high hydrophilic regions, as the antigenic sites tend to be exposed on the surfaces of proteins [29]. B cell epitopes are divided into two types: a conformational epitope, which is recognized by immune cells by its tertiary structure; and, a continuous epitope, which is recognized by its linear sequence of amino acids [30]. These epitopes are important for the determination of properties of peptides or proteins in vaccine development and diagnostics. Following the confirmation, additional PCR reactions were performed in order to amplify the gene. Each of the primers used was designed to carry specific restriction enzyme sites and it was expected that the PCR-amplified gene fragments would also contain these sites. Each of the gene fragments was designed to contain *Eco*RI restriction sites at the 5′ end of DNA fragments, which result in ‘sticky ends’ upon enzyme digestion. *Sna*BI restriction sites were introduced at the 3′ termini. This enzyme will leave ‘blunt end’ DNA fragments following digestion [16,31]. The ligation mixtures were then transformed into competent *E. coli* TOP10 cells. The pTrcHis2-NPt plasmid sequence contained the ampicillin-resistant gene [16]. This allowed for the selection of transformed cells in media containing ampicillin. 

The recombinant plasmid was successfully transformed into *E. coli* TOP10 cells. The positive transformants were chosen from ampicillin-resistant colonies of *E. coli* TOP10 cells and further analyzed by PCR while using forward primer of pTrcHis2 vector and reverse primers of respective inserts to confirm the correct orientation of the truncated VP1 inserts. The *E. coli* system was chosen for this study because post-translational processing, such as glycosylation, is not required for the synthesis of NPfl proteins [16,19]. In the present study, we showed that the expression levels of these proteins decreased after their expression peak. This was perhaps due to a degradation of the recombinant proteins inside the cells. Prolonged incubation was previously shown to induce the proteolysis of expressed proteins, leading to yield reduction [32].

Although the amino acid position 198–297 of VP1 protein mainly contains hydrophilic regions, this truncated VP1 polypeptide that fused to the protein carrier NPt was mainly expressed in inclusion bodies. Similar results were observed with the proteins, such as fragments, f, the Toxoplasma gondii rhoptry protein ROP2 fused with thioredoxin (TRX) or to the maltose-binding protein (MBP), which are well known for improving the solubility of fusion proteins [33]. It is known that the formation of inclusion bodies is affected by the rate of target protein translation in the bacterial host [34]. If the rate of protein expression is higher than the rate of their folding into secondary and tertiary structures, then they will likely be improperly folded. These proteins will then accumulate as insoluble aggregates in the bacterial cell [35] Thus, the recombinant NPt-VP1_198–297_ protein was purified under denaturing conditions. 

In the present study, the recombinant NPt-VPl_198–297_ was purified and immunized into an adult mouse. It was shown that the recombinant vaccine was able to elicit moderately high immune responses. Based on our findings, IgG was the most abundant form of immunoglobulin in serum. IgG plays an important role in complement activation and opsonization [36]. In most cases, high levels of IgG are formed after secondary immunization. It was also proven that the recombinants vaccine was able to elicit high immune response in adult mice [21]. This suggests that the NPt-VP1_198–297_ protein acted as a strong immunogen for the humoral response. When naive B-cells encounter specific antigen(s), they will rapidly divide and differentiate into immunoglobulin-producing plasma cells [37]. Consequently, greater magnitudes of antibody response were generated.

It had been shown that the antibodies elicited by VP1 proteins of EV71 were able to neutralize EV71 and inhibited the virus in order to infect the Vero or rhadobdomyosarcoma (RD) cells [38]. In our study, we showed that the anti-NPt-VP1_198–297_ antiserum prevented Vero cells from CPE at titer of 1:16 and the recombinant NPt-VP1_198–297_ was able to induce a significant neutralizing immune response against EV71. This result agreed with a previous finding by Xu et al. (2012), which purported that the neutralizing epitopes may locate at the amino acid 208–222 of VP1 proteins. The earlier study reported synthetic peptide, SP70, was able to elicit neutralizing antibodies in order to protect the RD cells from the infection of EV71 at titer of 1:32 [12]. VP1_1–100_ polypeptide, which was carried by NPt carrier (NPt-VP1_1–100_) in the prior art, had its efficacy as candidate vaccine tested and showed that it partially protected the neonates from EV71 infection, but has a lack of neutralizing epitope(s) [21]. The study of Ong and his colleagues (2010) addressed the correlation or neutralizing antibodies with the prevention of viral replication and spreading in skeletal muscle and central nervous system. Thus, this novel observation highlights the potential of NPt-VP1_198–297_ proteins as a candidate vaccine of EV71. Because lymphocyte proliferative responses are generally related to cell-mediated immunity [39] and its association with the viral clearance, we sought to investigate the T-cell responses after immunization with the candidate vaccine constructs. In the present study, based on the titer profiles of neutralizing antibody against EV71, increased levels of splenocyte proliferation in the vaccinated group were observed. According to Wu et al. (2001), the immunization of mice while using the full-length VP1 recombinant protein resulted in a mixed Th1 and Th2 response. Th1 and Th2 subsets of helper T-cells express distinct cytokine patterns, which reflect the different immune response pathways. Th1-cells are known to be involved in cell-mediated immunity, while Th2-cells function as helper T-cells in humoral immunity. A significantly higher titer (1:16) of NPt-VP1_198–297_ as compared to VP1fl (1:8) in the assay suggests that NPt-VP1_198–297_ promoted a better Th1 immune response when compared to VP1fl. The results suggested that this group was able to generate better immune responses, especially cell-mediated immunity as compared to the control group. The high titer of the serum shows a promising result on the capability of candidate vaccine in providing excellent immune stimulation. It was also suggested that splenocyte proliferation reaction is directly proportional to the cellular immune response that is important for viral killing [40].

Overall, the results from this study suggested that the NPt-VP1_198–297_ is an ideal candidate vaccine against EV71. A study by Wu et al. (2001) using a recombinant VP1 protein expressed in *E. coli* BL21 [8], showed that the VP1 with a complete adjuvant able to elicit a neutralizing antibody response, enhance T helper cell proliferation, and induce high levels of interleukin (IL)−10 and interferon (IFN)- gamma in mice. The findings from the study provide direct evidence that the VP1 contains neutralizing epitopes independent of other viral capsid proteins. This paves the way for the use of VP1 as a backbone antigen for developing subunit vaccines against EV71. IgG level increased in mice immunized with DNA vaccine; in contrast, this level declined after boosting immunization [41]. 

## 5. Conclusions

In conclusion, the findings from this study suggested that this protein construct of NPt-VP1_198–297_ is capable of eliciting EV71-specific neutralizing antibodies. At present, the recombinant NPt-VP1_198–297_ protein was expressed in an insoluble form and the purification of the recombinant protein was carried out in a denaturing condition, which often interferes with the quality of purified protein and increases the difficulties of the purification process. Additional studies for improving the purification process are currently being evaluated. Nevertheless, information from the current study has contributed towards further understanding NPt-VP1_198–297_ as a potential vaccine against EV71.

### Ethical Approval and Consent to Participate

All of the animal works in this study were approved by The Animal Care and Use Committee, Faculty of Veterinary Medicine, Universiti Putra Malaysia, Selangor (Code no: #10 R84). The animals were raised and cared for according to The Code to Care and Use of Animals in Research. The methods of animal experiments were performed under the guidelines that were prescribed by the Malaysian Association for Accreditation of Laboratory Animal Care.

## Figures and Tables

**Table 1:** Dilution of antibodies in 1X TBS.

Antibodies	Dilution in 1X TBS
Anti-Histidine	1:5000
Anti-NDV	1:9000
Anti-VP1	1:2000

**Table 2:** Titers profiles of neutralizing antibody against EV71.

Immunogen	Neutralization Titers
Anti-mouse adapted EV71 P5 serum	≥1:512
VP1fl	1:8
NPt-VP1_198–297_	1:16
NPfl	<1:8
Pre-immune serum	<1:8
